# Doublecortin in Oligodendrocyte Precursor Cells in the Adult Mouse Brain

**DOI:** 10.3389/fnins.2017.00143

**Published:** 2017-03-28

**Authors:** Jenna J. Boulanger, Claude Messier

**Affiliations:** School of Psychology, University of OttawaOttawa, ON, Canada

**Keywords:** myelin, adult neurogenesis, myelin remodeling, oligodendrocyte, plasticity, gliogenesis, cell migration

## Abstract

**Key Points**

- Oligodendrocyte precursor cells express doublecortin, a microtubule-associated protein.
- Oligodendrocyte precursor cells express doublecortin, but at a lower level of expression than in neuronal precursor.
- Doublecortin is not associated with a potential immature neuronal phenotype in Oligodendrocyte precursor cells.

Oligodendrocyte precursor cells (OPC) are glial cells that differentiate into myelinating oligodendrocytes during embryogenesis and early stages of post-natal life. OPCs continue to divide throughout adulthood and some eventually differentiate into oligodendrocytes in response to demyelinating lesions. There is growing evidence that OPCs are also involved in activity-driven *de novo* myelination of previously unmyelinated axons and myelin remodeling in adulthood. Considering these roles in the adult brain, OPCs are likely mobile cells that can migrate on some distances before they differentiate into myelinating oligodendrocytes. A number of studies have noted that OPCs express doublecortin (DCX), a microtubule-associated protein expressed in neural precursor cells and in migrating immature neurons. Here we describe the distribution of DCX in OPCs. We found that almost all OPCs express DCX, but the level of expression appears to be much lower than what is found in neural precursor. We found that DCX is downregulated when OPCs start expressing mature oligodendrocyte markers and is absent in myelinating oligodendrocytes. DCX does not appear to signal an immature neuronal phenotype in OPCs in the adult mouse brain. Rather, it could be involved either in cell migration, or as a marker of an immature oligodendroglial cell phenotype.

## Introduction

Although traditionally perceived as non-regenerative tissue, the adult brain does retain the ability to generate new neurons, as first described by Messier (Messier et al., 1958) and confirmed by Altman and Das (1964). More specifically, studies have determined that neurogenesis in the adult rodent brain takes place in two distinct regions: the subgranular layer of the dentate gyrus of the hippocampus, with newly generated neurons migrating to the granular layer of the dentate gyrus (Kaplan and Bell, 1984; Stanfield and Trice, 1988; Cameron et al., 1993; Kuhn et al., 1996) and in the subventricular zone of the lateral ventricles, from where the newly generated neurons migrate to the olfactory bulbs using a pathway known as the rostral migratory stream (Lois and Alvarez-Buylla, 1994; Alvarez-Buylla and Garcıa-Verdugo, 2002).

The migration of these newly generated neuroblasts to their final destination is thought to be facilitated by the expression of doublecortin (DCX), a protein that participates in the polymerization of microtubules (Francis et al., 1999; Gleeson et al., 1999). DCX is only transiently expressed in proliferating progenitor cells and in newly generated neuroblasts and its expression is downregulated as the cells begin to express markers of a mature neuronal state. This has led to the use of DCX as a selective marker of adult neurogenesis (Brown et al., 2003). However, DCX expression is not restricted to the dentate gyrus or areas involved in the addition of new neurons to the olfactory bulbs (Nacher et al., 2001; Dayer et al., 2005; Luzzati et al., 2006; Xiong et al., 2008; Cai et al., 2009; Klempin et al., 2011; Werner et al., 2012; Saul et al., 2014). Some authors have also observed DCX in cells expressing immature neurons markers located in the layer III of the piriform cortex and endopiriform nucleus of adult rodents which do not appear to be migrating neurons (Rivers et al., 2008; Guo F. et al., 2010; Klempin et al., 2011; Clarke et al., 2012). Finally, as we show here, DCX is widely expressed in oligodendrocyte precursor cells (OPCs; also known as NG2-glial cells, synantocytes, or polydendrocytes; Tamura et al., 2007a; Ehninger et al., 2011).

OPCs are a type of glial cell that give rise, as their name suggests, to myelinating oligodendrocytes during embryogenesis and early stages of post-natal life (Nishiyama et al., 2002). However, a large number of OPCs maintain their undifferentiated state after these initial developmental stages and OPCs are thus abundant in the adult brain, corresponding to ~5–8% of the total cell population (Dawson et al., 2003; Gallo et al., 2008). Adult OPCs form non-overlapping fields that are uniformly distributed between the gray and white matter of the central nervous system (Dawson et al., 2003; De Biase et al., 2010; Kukley et al., 2010; Hughes et al., 2013). While their proliferative activity does decline with age, they continue to undergo cell division throughout adulthood, representing the most active population of cycling cells within the adult brain (Dawson et al., 2003; Psachoulia et al., 2009). The fate of these adult-generated OPCs has not been clearly established (Boulanger and Messier, 2014). While most daughter cells appear to maintain an OPC phenotype, it has been demonstrated that a subset of these cells differentiates into a mature myelinating oligodendroglial phenotype (Dimou et al., 2008; Kang et al., 2010; Clarke et al., 2012). Furthermore, some researchers have reported that postnatal OPCs are multipotent and have the capacity to differentiate into astrocytes and neurons in multiple regions of the adult CNS (Belachew et al., 2003; Aguirre and Gallo, 2004; Aguirre et al., 2004; Dayer et al., 2005; Tamura et al., 2007a; Rivers et al., 2008; Zhu et al., 2008; Guo F. Z. et al., 2009, 2010; Robins et al., 2013). However, this ability to differentiate into neurons is not supported by other studies (Dimou et al., 2008; Komitova et al., 2009; Kang et al., 2010; Richardson et al., 2011; Zhu et al., 2011; Nishiyama et al., 2016). This remains a debated point at this time (Larson et al., 2016; Nishiyama et al., 2016; Viganò and Dimou, 2016).

Here, we show that despite evidence for multipotency in OPCs, DCX is not associated with an immature neuronal phenotype in these cells. Rather, it could be involved either in cell migration, or as a marker of an immature oligodendroglial cell phenotype.

## Materials and methods

### Animals

Animals were 4–5 month old Long-Evans rats (Charles River, St-Constant, Qc, Canada), 5–10 weeks old CD-1 mice (Charles River, St-Constant, Qc, Canada), and 3–5 month-old C57BL/6J mice (Jackson Laboratory, Bar Harbor, Maine, USA) All the animals used in this study were individually housed in a 21 ± 1°C vivarium, maintained on a 12-h light/dark cycle, and had *ad libitum* access to food and water. All animal procedures were done in accordance with the recommendations of the Canadian Council on Animal Care and were approved by the Animal Care Committee of the University of Ottawa.

### Transgenic animals

NG2CreER BAC transgenic mice (Jackson Laboratory, Bar Harbor, Maine, USA; described in Zhu et al., 2011) were bred in house with R26-stop-EYFP transgenic mice on a C57BL/6J background (Jackson Laboratory, Bar Harbor, Maine, USA) to generate the NG2-CreER:EYFP reporter mouse. Three to five-month old animals (*n* = 8) received i.p injections of 6 mg of tamoxifen per day, over a period of 5 days. Six days after the last tamoxifen injection, the animals' drinking water was replaced with 5-bromo-2′-deoxyuridine (BrdU) in sweetened water (100 ml water + 0.125 g saccharine + 3 g dextrose + 0.1 g BrdU) for a total of 8 days. Mice were perfused 8 weeks following the end of BrdU exposure.

### Tissue processing

Anesthetized rats or mice were transcardially perfused with saline followed by Lana's fixative (4% paraformaldehyde-picric acid; modified from Zamboni and Demartin, 1967). Brains were post-fixed in this fixative for 1 h before being incubated in 10% sucrose in sodium phosphate buffer (0.1 M, pH 7.2) overnight at 4°C. Brains were then frozen using CO_2_ and cut in 14 μm sagittal sections using a cryostat.

### Immunochemistry

#### Peptide competition assay

To determine the specificity of the goat anti-DCX antibody (Santa Cruz, sc-8066) and the guinea-pig anti-DCX antibody (Chemicon, AB5910) a peptide competition assay was performed. The DCX peptide was no longer available at Chemicon. As such, both antibodies were submitted to a competition assay with the DCX peptide provided by Santa Cruz Biotechnology (sc-8066). A 1:5 solution of anti-DCX antibody and anti-DCX peptide (sc-8066P, Santa Cruz) was incubated at room temperature for 1 h. The preparation was then diluted in 0.3% Triton and each section was covered with 50 μl and incubated at room temperature for 3 h. Following incubation, slides were washed successively three times for 5 min in PBS (10 mM). Each section was then incubated for 30 min at 37°C with 50 μl of the secondary antibody Alexa488 donkey anti-Goat (1:1,000, Invitrogen) or Alexa488 anti-guinea pig (1:500, Jackson Immuno Research) diluted in 0.3% Triton in 10X PBS. After incubation, slides were washed successively three times for 5 min in 10X PBS. Sections were imbedded in custom-made anti-fade solution (p-Phenylenediamine and glycerol in PBS solution) and cover-slipped with micro cover glasses (VWR Scientific).

#### Primary antibodies

Antibodies used and dilutions are presented in Table 1. Primary antibody solutions were diluted in 0.3% Triton in 10X PBS. Tissue sections were covered with 50 μl of the primary antibody solution and parafilm was placed on top to prevent evaporation. Slides were incubated at room temperature in a humidified chamber for 3 h. Following incubation, slides were washed successively three times for 5 min in 10X PBS. An anti-GFP antibody that also recognize EYFP was used to label the NG2-CreER:EYFP-positive OPCs because it improved visualization of the EYFP NG2 reporter.

**Table 1:** **List of antibodies used**.

**Primary antibodies**			
**Host**	**Target**	**Concentration**	**Company**
Rabbit	GFP (EYFP)	1/1,000	Abcam (ab290)
Rabbit	PDGFR_α_	1/250	Santa Cruz (sc-338)
Rat	PDGFR_α_	1/500	Abcam (ab93531)
Rabbit	GST_π_	1/500	MBL (311-h)
Goat	DCX	1/100	Santa Cruz (sc-8066)
Guinea Pig	DCX	1/500	Chemicon (AB5910)
Rabbit	DCX	1/1,000	Abcam (Ab18723)
Mouse	Rbfox3 (NeuN)	1/500	Millipore (MAB377)
Goat	Sox10	1/500	Santa Cruz (sc-17342)
Rat	BrdU	1/500	Abcam (ab6326)
Mouse	OLIG1	1/1,000	Millipore (MAB5540)
Donkey	Anti-Rabbit Alexa 488	1/1,000	Invitrogen
Donkey	Anti-Goat Alexa 488	1/1,000	Invitrogen
Donkey	Anti-Guinea Pig Alexa 488	1/500	Jackson immuno research
Donkey	Anti-Rabbit Alexa 594	1/1,000	Invitrogen
Donkey	Anti-Mouse Alexa 594	1/1,000	Invitrogen
Donkey	Anti-Goat Alexa 594	1/1,000	Invitrogen
Donkey	Anti-Rat Alexa 594	1/1,000	Invitrogen
Donkey	Anti-Rabbit Alexa 680	1/500	Invitrogen
Donkey	Anti-Mouse Alexa 680	1/500	Invitrogen
Donkey	Anti-Guinea Pig Alexa 680	1/500	Jackson immuno research
Donkey	Anti-Rat Alexa 680	1/500	Abcam

#### Secondary antibodies

Antibodies used and dilutions are presented in Table 1. Secondary antibody solutions were diluted in 0.3% Triton in 10X PBS. Tissue sections were covered with 50 μl of the secondary antibody solution and parafilm was placed on top to prevent evaporation. Slides were incubated in a humidified chamber for 30 min at 37°C. Following incubation, slides were washed successively three times for 5 min in 10X PBS.

#### Cell nuclei staining

Cell nuclei were stained using the DNA stain Hoechst 33342 (Invitrogen). The Hoechst dye was diluted in 0.3% Triton in 10X PBS to yield a final concentration of 1:20,000. Each section was covered with 100 μl of this solution and was left to incubate for 10 min in a humidified chamber at room temperature. Slides were washed successively three times for 5 min in 10X PBS. Sections were imbedded in custom-made anti-fade solution (p-Phenylenediamine and glycerol in PBS solution) and cover-slipped.

#### BrdU immunostaining

To preserve DCX and other immunostaining during the acid/heat denaturation step required for BrdU labeling, a previously described protocol was used (Boulanger et al., 2016). Immunohistochemistry for DCX and GFP were conducted first. This was followed by an overnight post-fixation step where slides were incubated in a humid chamber with Lana's fixative overnight at 4°C. Slides were then rinsed in 10X PBS 3 times for 5 min. This post-fixation step allowed the preservation of the DCX and GFP immunohistochemistry during the acid/heat denaturation step required for BrdU labeling. This was followed by DNA denaturation, where slides were incubated in HCl 2N for 30 min at 37°C. Slides were then rinsed 3 times for 5 min in a 0.1M borate buffer pH 8 and 3 times in 10X PBS for 5 min. For BrdU immunohistochemistry, slides were incubated in a humid chamber with a rat anti-BrdU antibody dissolved in PBS with 0.3% Triton-X in the dark for 3 h at room temperature. Finally, slides were incubated in a humid chamber with a donkey anti-rat secondary antibody dissolved in PBS with 0.3% Triton-X for 30 min at 37°C. Slides were then rinsed in PBS 3 times for 5 min, treated with custom-made anti-fade solution (p-Phenylenediamine and glycerol in PBS solution) and cover-slipped.

#### Microscopy

Immunofluorescence results were visualized using an Olympus BX51 fluorescence microscope (Olympus Corporation, Tokyo, Japan) attached to a ProgRes MF Scan camera (Jenoptik, Jena, Thuringe, Germany). Digital images were captured using the ProgRes CapturePro 2.5 software (Jenoptik, Jena, Thuringe, Germany). High-resolution observations were carried out on an Olympus FV1000 BX61 laser scan confocal microscope (Olympus Corporation, Tokyo, Japan) and images were captured using the Olympus Fluoview software (Olympus Corporation, Tokyo, Japan).

## Results

### Specificity of the weak DCX staining in OPC

**Figure 1:**
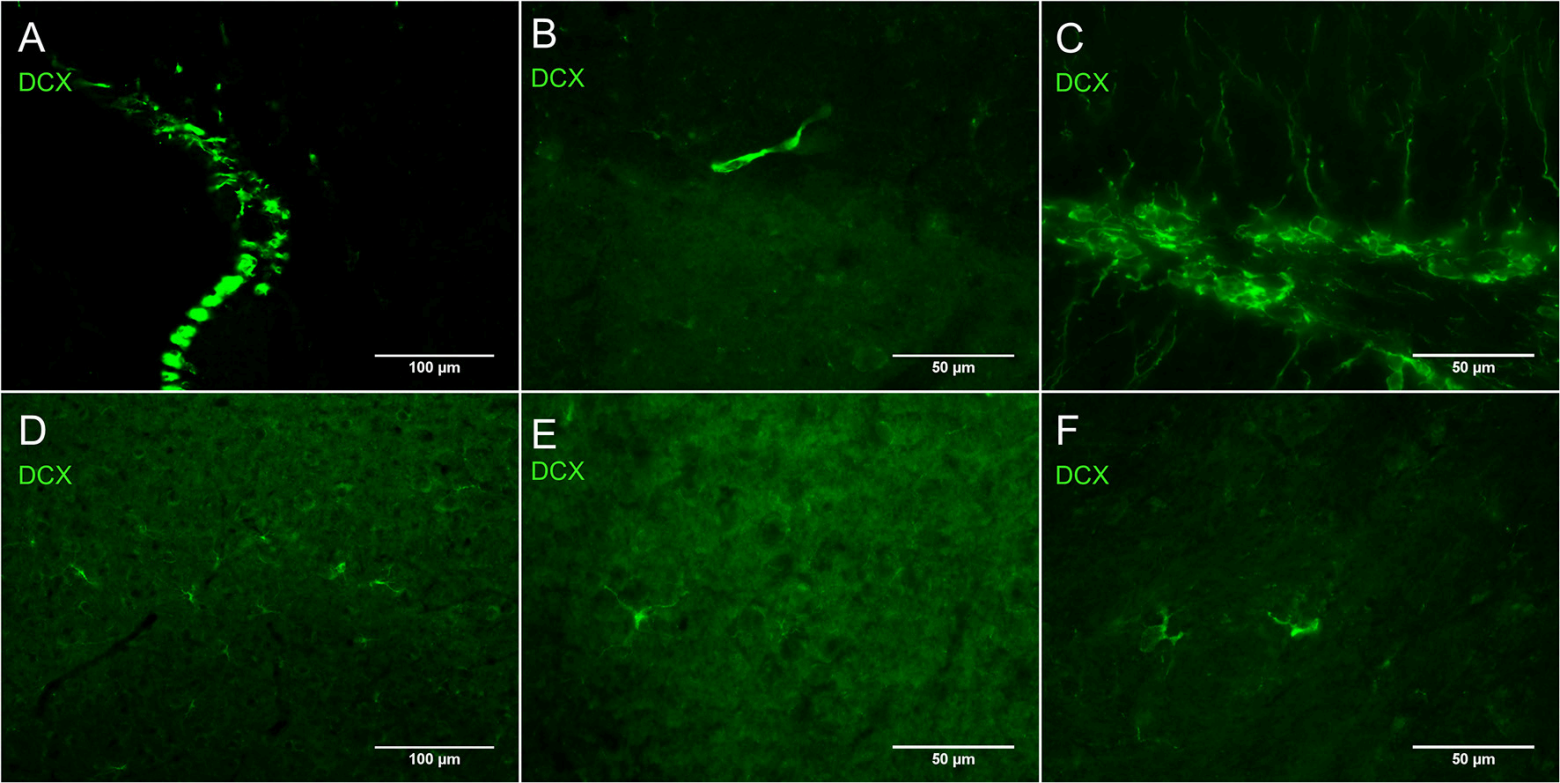
**Intensity of DCX immunostaining in neurogenic (A–C)** and in non-neurogenic zones **(D–F)** using guinea pig anti-DCX and Alexa 488 anti-guinea pig antibodies. Exposure times for DCX labeling in non-neurogenic areas were in general double those of neurogenic areas. Pictures taken with a fluorescence microscope in the subventricular zone (**A**, 20X), rostral migratory stream (**B**, 40X), subgranular zone of the dentate gyrus (**C**, 40X), cortex (**D**, 20X), cortex (**E**, 40X), and striatum (**F**, 40X).

Figure 1 shows the difference in relative intensity of staining of cells in neurogenic areas (Figures 1A–C) and non-neurogenic areas (Figures 1D–F). All pictures were taken with the same objectives, the same gain but exposure times were generally twice as long for non-neurogenic DCX labeling. Although, we have observed this weak DCX staining with different immunohistochemistry protocol variants in rat and mouse tissue, the best results appear to be dependent on a short 1-h post-fixation period.

**Figure 2:**
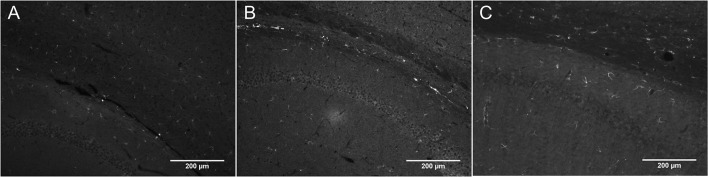
**DCX immunostaining in corpus callosum and hippocampus of rat (A)** and mouse brain **(B,C)** at 10X with a fluorescence microscope using a variety of anti-DCX antibodies. Goat anti-DCX from Santa Cruz (**A**, 1/100 in PBS-Triton), guinea pig anti-DCX from Chemicon (now Millipore; **B**, 1/500 in PBS-Triton), and rabbit anti-DCX from Abcam (**C**, 1/1000 in PBS-Triton).

**Figure 3:**
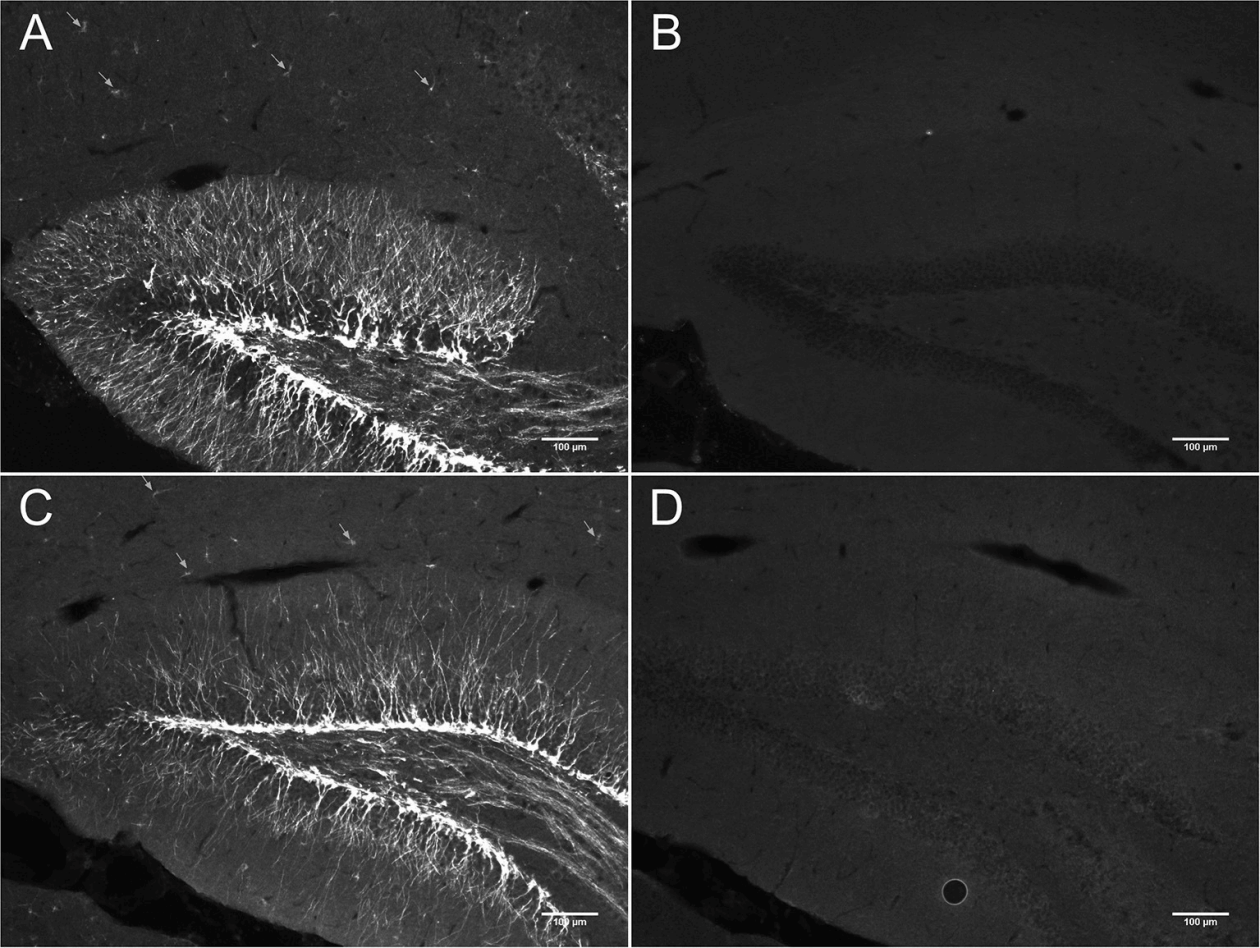
**DCX immunostaining in hippocampus of mouse brain with (B,D)** and without anti-DCX peptide **(A,C)**. Pictures were taken at 10X using a fluorescence microscope. Goat anti-DCX from Santa Cruz Biotechnology was used in images **(A,B)** and guinea pig anti-DCX from Chemicon (now Millipore) was used in images **(C,D)**. Arrows show DCX staining in OPCs located outside of the known neurogenic zone that is the dentate gyrus of the hippocampus.

Figure 2A shows DCX immunostaining using a goat anti-DCX from Santa Cruz (SC-8066) used by many researchers. Figures 2B,C show DCX immunostaining using respectively a guinea pig anti-DCX from Chemicon (AB5910) and a rabbit anti-DCX from Abcam (Ab18723). These observations suggest that light DCX cell labeling outside of neurogenic zones is not specific to one primary antibody. In general, the original guinea pig anti-DCX from Chemicon (AB5910) produced brighter labeling but with slightly increased background. Because others and we typically use the Santa Cruz DCX primary antibodies, we also confirmed its specificity through the absence of staining after blocking the DCX primary antibody with the corresponding DCX immunizing peptide (Santa Cruz SC-8066P) that we also used to block the guinea pig anti-DCX (Figure 3). The absence of labeling observed in the presence of the immunizing peptide indicated that the primary anti-DCX antibody recognizes DCX protein. Figure 3 also shows the relative intensity of DCX in OPCs compared to the cells in the subgranular layer of the dentate gyrus. Together, these observations confirm that the weak DCX staining observed in OPCs throughout the mouse and rat brain is a specific and reproducible finding.

**Figure 4:**
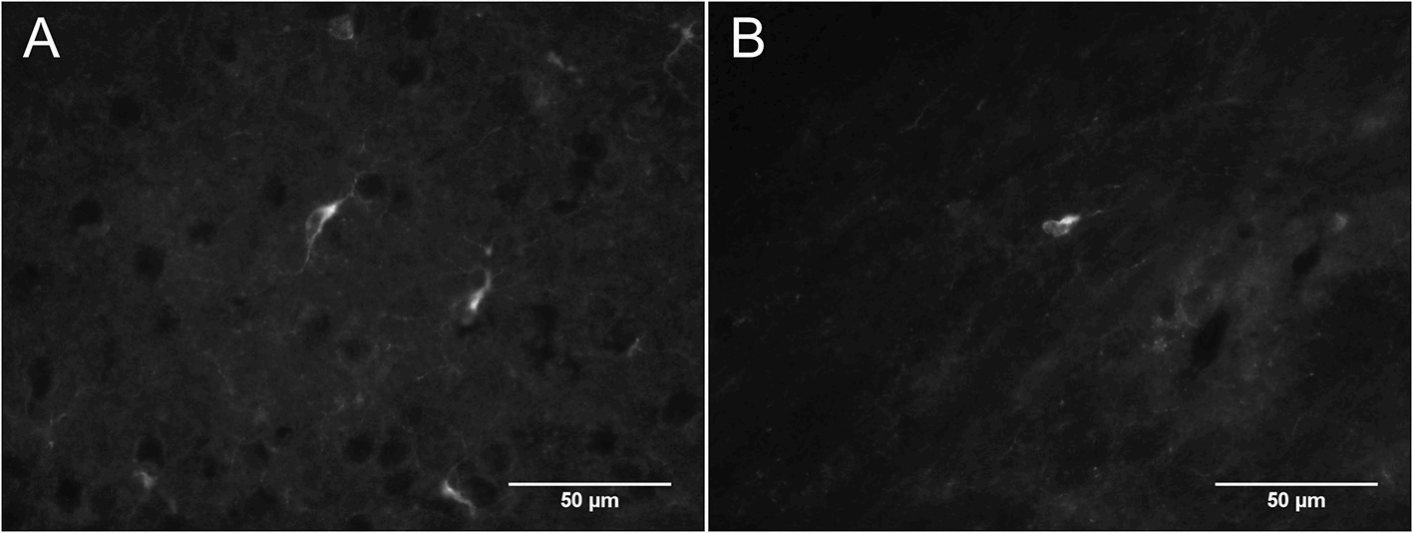
**(A,B)** Two examples of DCX-positive cells outside of neurogenic zones: DCX immunostaining (guinea pig anti-DCX) is concentrated in one pole of the cell. Pictures were taken in the cortex at 40X with a fluorescence microscope.

**Figure 5:**
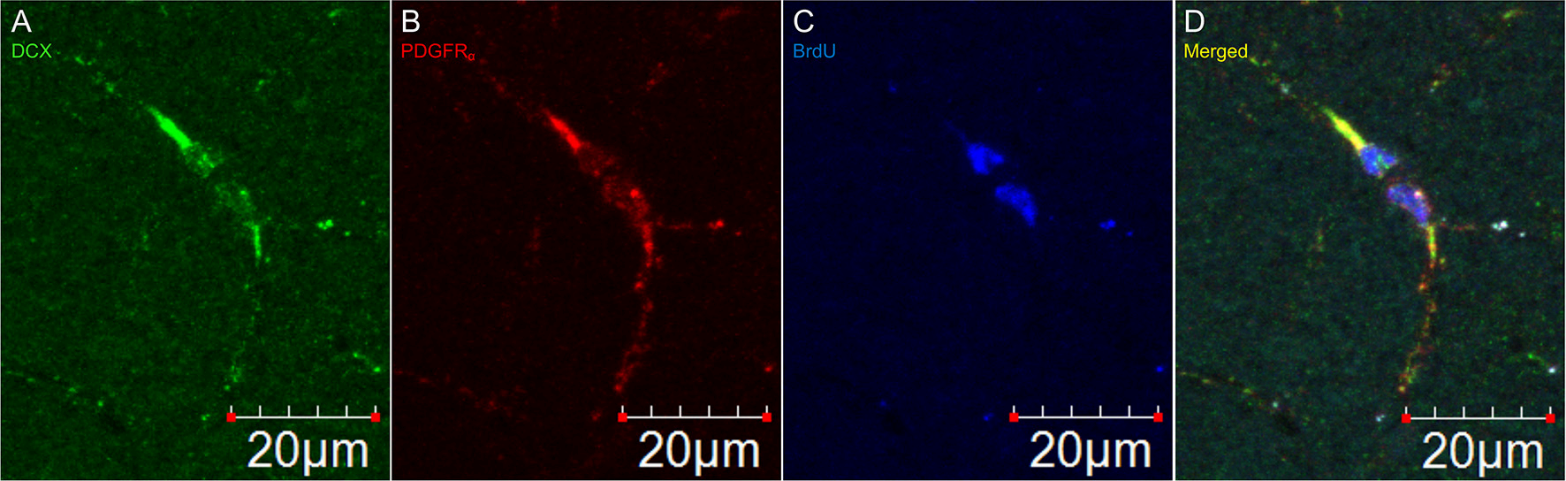
**DCX immunostaining appears after division of an OPC**. Pictures taken in the cortex at 60X with a laser scan confocal microscope. DCX in green **(A)**, PDGFR_α_ in red **(B)**, BrdU in blue **(C)**, and merged **(D)**.

In DCX lightly stained cells, there is a characteristic higher intensity staining of one pole of the cell: this is very typical of the DCX in OPCs. The rest of the cell body is weakly stained while the membrane appears slightly more stained (Figure 4). The most intense staining is found at the hillock of the main branch of the cell processes. Most of the cell processes also appear to contain DCX with weaker staining being found in the finer processes. Most cells that are weakly stained for DCX are multipolar with a minority of cells that are bipolar or, more rarely, unipolar. In DCX-labeled cell with a bipolar form, only one of the poles had intense DCX labeling, usually the one with the most processes. In instances where OPCs were newly divided, as demonstrated by PDGFR_α_ labeling coupled with BrdU immunostaining, we observed early DCX labeling (Figure 5).

### Identity of cells labeled with DCX outside neurogenic areas

**Figure 6:**
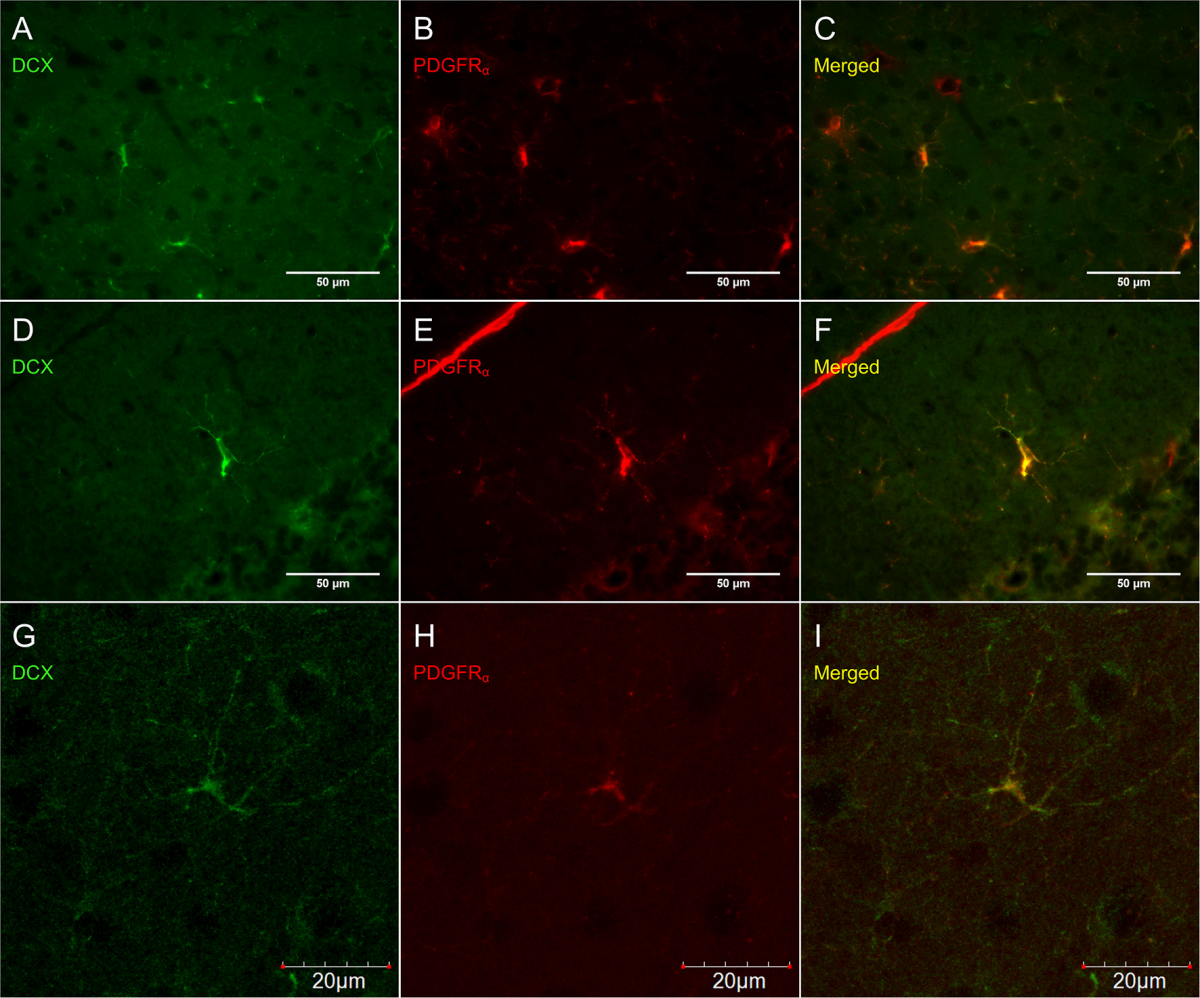
**DCX immunostaining (guinea pig anti-DCX) outside of neurogenic zones is co-localized with PDGFR_α_ (rat anti-PDGFR_α_), an OPC-marker**. Pictures taken at 40x with a fluorescence microscope in the cortex **(A–C)** and in the cerebellum **(D–F)** and at 40x with a laser scan confocal microscope in the cortex **(G–I)**. DCX in green **(A,D,G)**, PDGFR_α_ in red **(B,E,H)**, and merged **(C,F,I)**.

**Figure 7:**
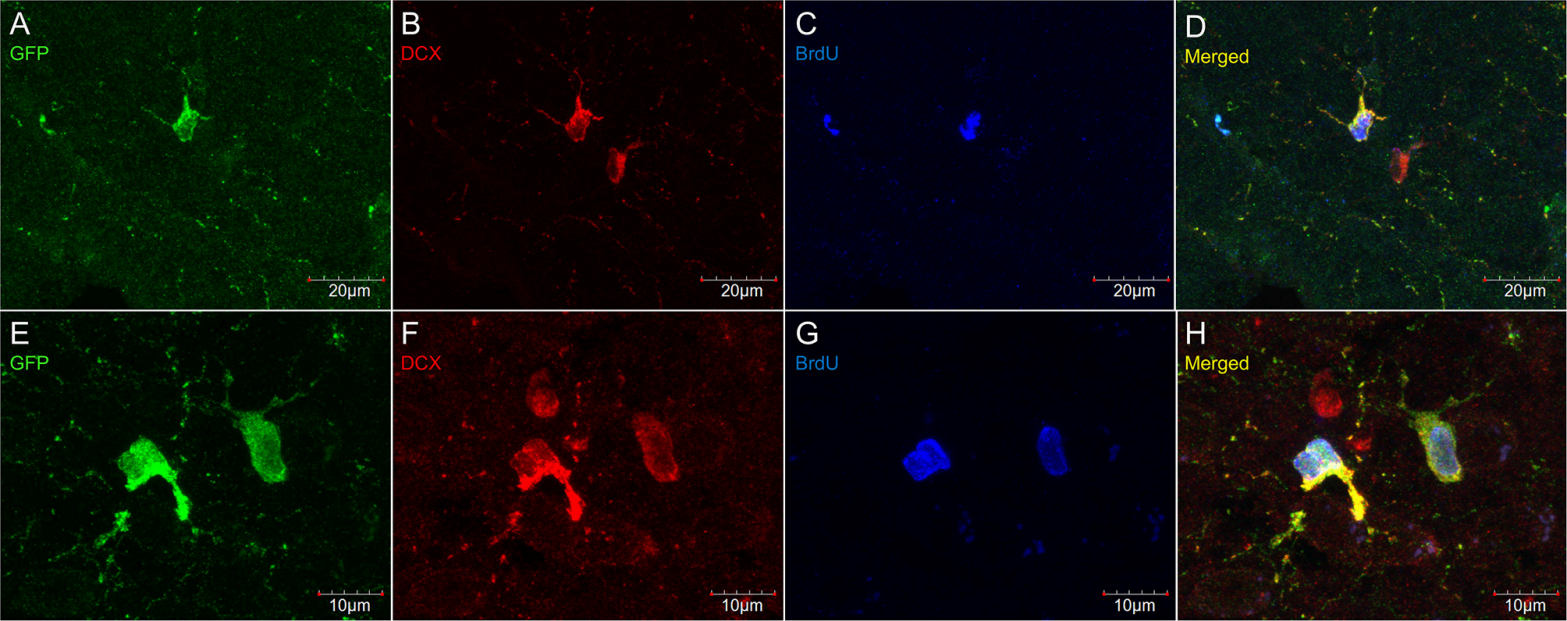
**DCX immunostaining (goat anti-DCX) in OPC-like cells outside of neurogenic zones is co-localized with GFP in NG2-CreER:EYFP reporter mice**. Pictures taken in the cortex at 40X with a laser scan confocal microscope **(A–D)**. Pictures taken in the cortex at 60X with a laser scan confocal microscope **(E–H)**. GFP in green **(A,E)**, DCX in red **(B,F)**, BrdU in blue **(C,G)**, and merged **(D,H)**.

Virtually all DCX weakly stained cells were also labeled with PDGFR_α_, an OPC marker (Figure 6). These double-labeled cells are observed throughout the brain and follow the usual distribution of OPCs. DCX lightly-labeled OPC-like cells outside of neurogenic zones are co-localized with GFP in NG2-CreER:EYFP reporter mice (Figure 7). When tamoxifen is intraperitoneally injected into these mice, it induces a Cre-mediated recombination of the floxed sequences and EYFP expression is thus observed in NG2-positive cells. Since NG2 is also a marker of OPCs, this confirms that OPCs express DCX at least at some point in time, including when they have proliferated, as demonstrated by BrdU immunostaining (Figure 7).

**Figure 8:**
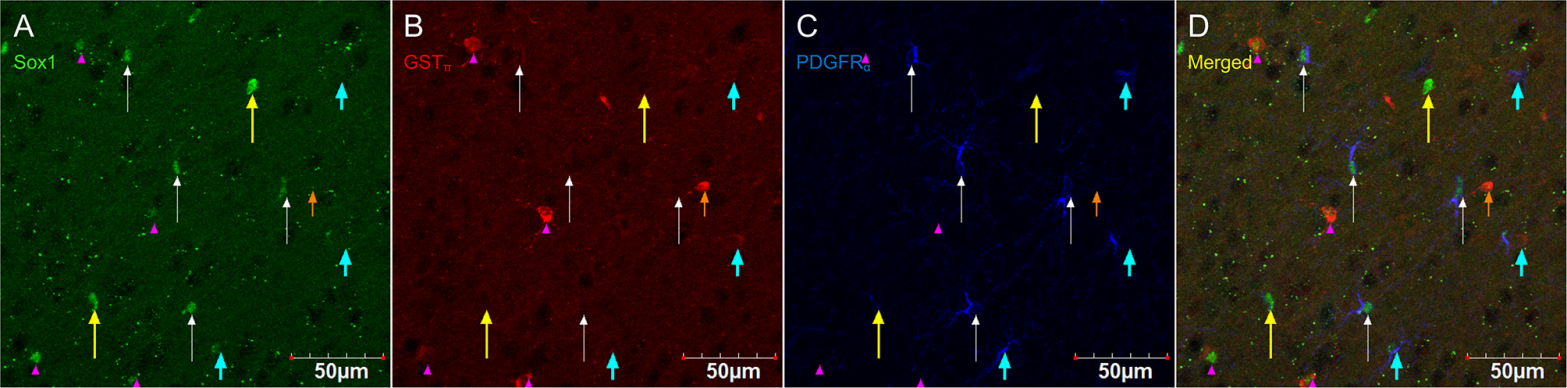
**PDGFR_α_-positive OPC immunostaining (rat anti-PDGFR_α_) are sometimes co-localized with the immature oligodendrocyte marker Sox10, but never with the marker of mature oligodendrocytes GST_π_**. Pictures taken in the cortex at 40X using a laser scan confocal microscope. Sox10 in green **(A)**, GST_π_ in red **(B)**, PDGFR_α_ in blue **(C)**, and merged **(D)**. Sox10-positive/GST_π_-negative/PDGFR_α_-negative cell (long yellow arrow), Sox10-negative/GST_π_-positive/PDGFR_α_-negative cell (short and narrow orange arrow), Sox10-negative/GST_π_-negative/PDGFR_α_-positive cell (short and thick aqua arrow), Sox10-positive/GST_π_-positive/PDGFR_α_-negative cell (long and narrow white arrow), and Sox10-positive/GST_π_-negative/PDGFR_α_-positive cell (pink arrow head).

**Figure 9:**
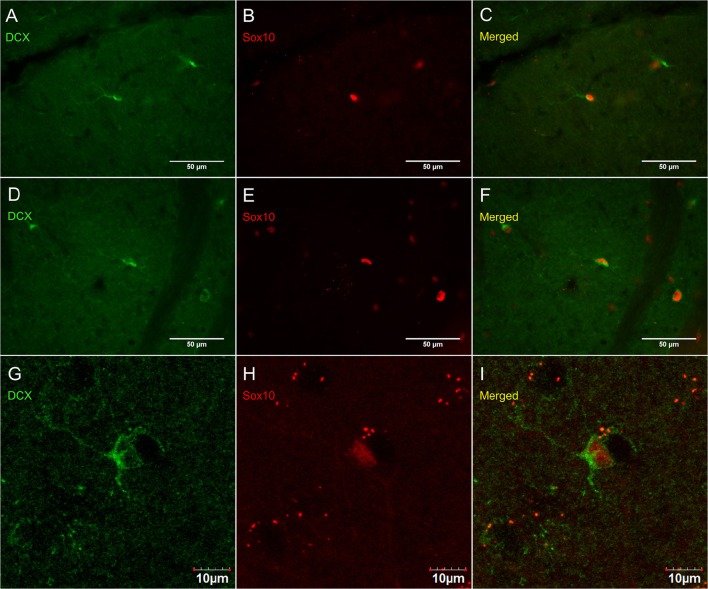
**DCX immunostaining (guinea pig anti-DCX) outside of neurogenic zones is sometimes co-localized with Sox10, a marker of immature oligodendrocytes**. Pictures were taken at 40X using a fluorescence microscope with a fluorescence microscope in the cerebellum **(A–C)** and corpus callosum **(D–F)**. Pictures were taken at 40X using a laser scan confocal microscope in the cortex **(G–I)**. DCX in green **(A,D,G)**, Sox10 in red **(B,E,H)**, and merged **(C,F,I)**.

**Figure 10:**
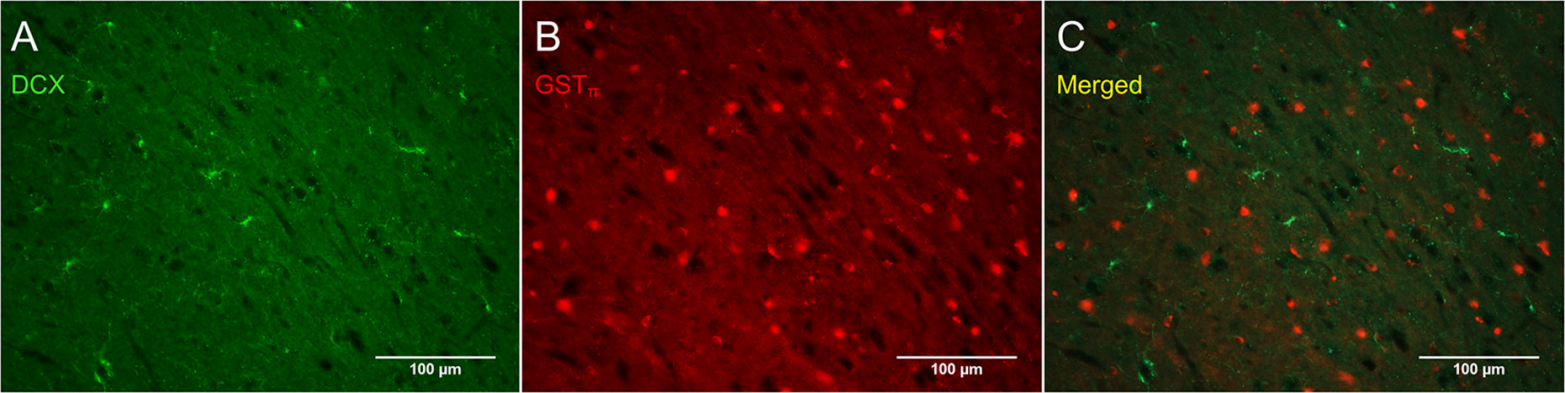
**DCX immunostaining (guinea pig anti-DCX) outside of neurogenic zones is not co-localized with GST_π_, a marker of mature oligodendrocytes**. Pictures were taken in the cortex at 20X with a fluorescence microscope. DCX in green **(A)**, GST_π_ in red **(B)**, and merged **(C)**.

Figure 8 shows that PDGFR_α_-positive OPC immunostaining are sometimes co-localized with the immature oligodendrocyte marker Sox10 (Stolt et al., 2002), but never with the marker of mature oligodendrocytes GST_π_ (Deloulme et al., 2004; Polito and Reynolds, 2005; Nishiyama, 2007; Taupin, 2010). Similarly, DCX immunostaining outside of neurogenic zones are sometimes co-localized with Sox10 (Figure 9) but not with GST_π_ (Figure 10). Sox10 regulates myelin gene expression in oligodendrocytes and is therefore expressed by OPCs that are transitioning to a mature, myelinating oligodendroglial phenotype (Stolt et al., 2002; Liu et al., 2007). During that transition, some, but not all, adult-brain OPCs express Sox10 and conversely, some, but not all oligodendrocytes, express Sox10.

**Figure 11:**
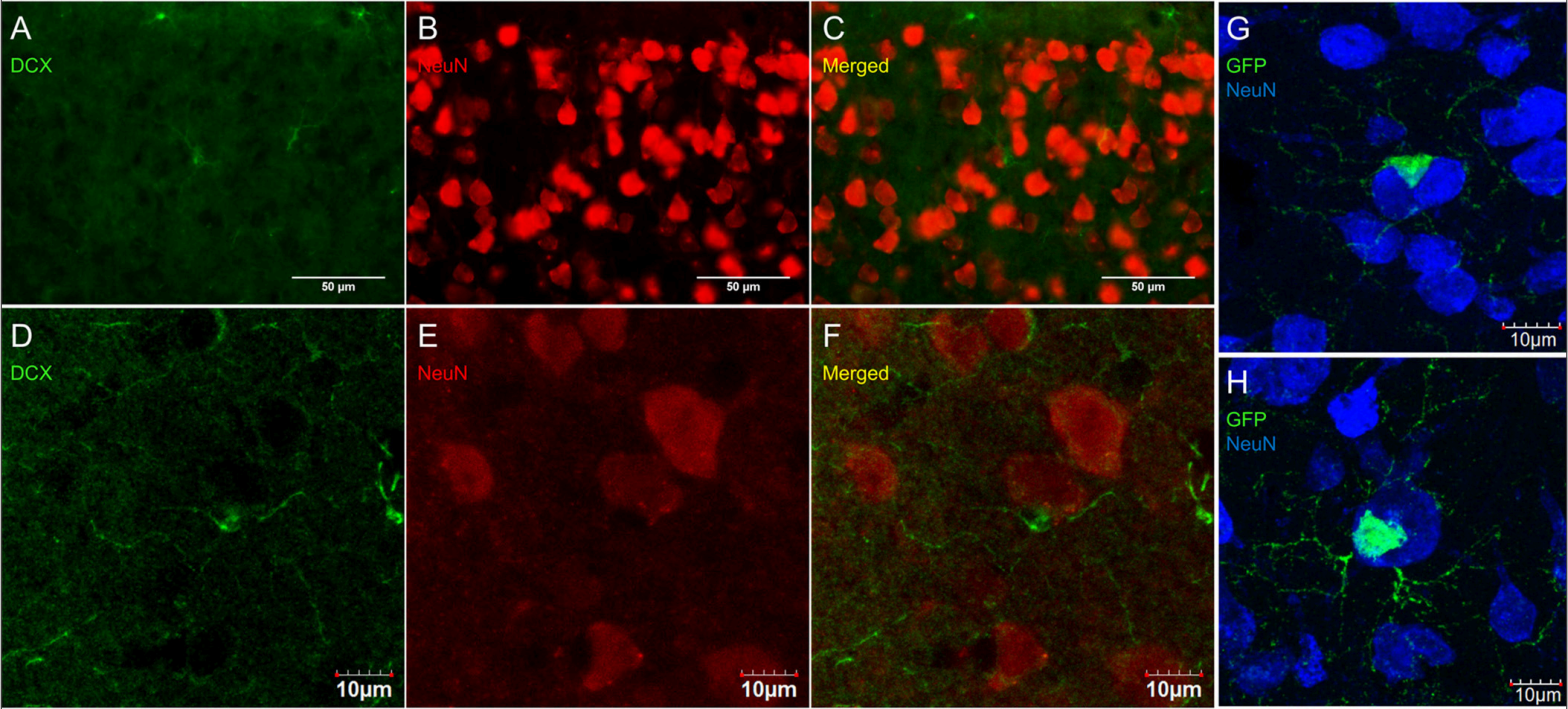
**OPCs form close contacts with neurons, but these cells are not co-localized**. Pictures taken in the cortex of CD-1 mice at 40X using a fluorescence microscope **(A–C)** and a laser scan confocal microscope **(D–F)**. DCX (guinea pig anti-DCX) in green **(A,D)**, NeuN in red **(B,E)**, and merged **(C,F)**. Pictures taken in the cortex of NG2-CreER:EYFP reporter mice at 60X using a laser scan confocal microscope with GFP in green and NeuN in blue **(G,H)**.

Some researchers have reported DCX labeling outside of neurogenic zones together with labeling for neuronal markers (for example NeuN; Nacher et al., 2001; Dayer et al., 2005; Luzzati et al., 2006; Xiong et al., 2008; Cai et al., 2009; Klempin et al., 2011; Werner et al., 2012; Saul et al., 2014). We have observed numerous instances of closely apposed OPC-neuron pairs (Figures 11C,F,G) that can appear in some instances as double-labeled cells (Figure 11H). These were found in outbred mice (CD-1; Figures 11A–F) as well as in the NG2-CreER:EYFP reporter mice (Figures 11F,G). The occurrence of OPC-neuron pairs has been mentioned previously (Butt et al., 2002; Sakry et al., 2011).

## Discussion

In the present report, we found that most OPCs express low levels of DCX in all parts of the brain where these cells are found. Low levels of DCX protein in OPCs is better visualized in lightly fixed brain tissue. The intensity of labeling also varies between commercially available DCX antibodies. Although, we did not determine when exactly DCX starts being expressed in OPCs after division, the observation that virtually all OPCs appear to express DCX suggest that, in the adult rodent brain, OPCs express DCX sometime after division and continue to express DCX until they differentiate into myelinating oligodendrocytes. This conclusion is supported by the sparse number of OPCs that express both DCX and the transcription factor Sox10, which is crucial for the final transformation of OPCs into myelinating oligodendrocytes (Stolt et al., 2002), and the absence of co-labeling of DCX together with GST_π_, a marker of mature myelinating oligodendrocytes (Tansey and Cammer, 1991; Tamura et al., 2007b; Simon et al., 2011).

Classically, DCX has been described as a selective marker of adult neurogenesis (Brown et al., 2003). As such, the observation of DCX-positive cells in various parts of the mammalian CNS previously led to the suggestion that new neurons are produced outside of the dentate gyrus and the subventricular zone (Dayer et al., 2005; Tamura et al., 2007a). In support of this hypothesis came reports that NG2-positive precursors are multipotent and can generate functional neurons (Belachew et al., 2003; Aguirre and Gallo, 2004; Aguirre et al., 2004; Dayer et al., 2005; Tamura et al., 2007a; Rivers et al., 2008; Zhu et al., 2008; Guo F. Z. et al., 2009, 2010; Robins et al., 2013).

Because of these reports, we examined if DCX-positive found outside of neurogenic zones also express mature or immature neuronal markers. We examined patterns of labeling using antibodies against Pax6—a paired box gene which is expressed by immature glutamatergic neurons (Bayatti et al., 2008), Pax2—a paired box gene expressed in GABAergic neurons (Batista and Lewis, 2008), and HuCD and Rbfox3 (NeuN)—markers of mature neurons. We found some very rare examples of co-labeling of lightly stained DCX cells either with Pax2, Rbfox3, or HuCD but that did not express OPC markers such as PDGFR_α_. Sometimes the closely apposed OPC-neuron pairs could be interpreted as double-labeled cells. In these instances, an OPC and a neuron could be superimposed in the field of view, as shown in Figure 11. While these observations do not rule out the possibility that OPCs can generate mature neurons, it may help explain the conclusion drawn by others that all DCX-positive cells, including those located outside of the dentate gyrus and subventricular zone, have neuronal attributes. These observations do not rule out the possibility of OPC-derived neurogenesis but they suggest additional caution to exclude other possibilities (see further discussion of this issue in Dimou and Gallo, 2015; Feliciano et al., 2015).

Finally, the question remains as to the role of doublecortin in OPCs. Since DCX is a microtubule-associated protein and because DCX-positive cells outside of neurogenic zones do not co-express mature neuronal markers, it is unlikely to be associated with a potential for OPCs to differentiate into neurons. It may, however, be involved in their migration over small distances as they monitor a unique territory that is not shared by other OPCs (Hughes et al., 2013). Furthermore, recent studies in missense DCX gene expression suggest a role in tubulin organization that could be associated with migration but also with process extension to establish and remodel the synaptic connections between neurons and OPCs (Tsai et al., 2016). This is significant since OPCs are known to form glutamatergic and GABAergic synapses with neurons (Bergles et al., 2000; Lin and Bergles, 2004). Therefore, this report as well as others suggest that it is time to stop viewing DCX as a marker of newly generated neurons but, rather, as a marker of cells that are undergoing migration or other forms of process reorganization.

## Author contributions

JJB and CM contributed equally to the design analysis and preparation of the manuscript.

### Conflict of interest statement

The authors declare that the research was conducted in the absence of any commercial or financial relationships that could be construed as a potential conflict of interest. The reviewer CM and handling Editor declared their shared affiliation, and the handling Editor states that the process nevertheless met the standards of a fair and objective review.

## Abbreviations

DCX: Doublecortin
GSTπ: glutathione S-transferase pi
NG2: chondroitin sulfate proteoglycan neuron-glia antigen 2
Olig1: bHLH transcription factor 1
OPC: oligodendrocyte precursor cell
PDGFRα: platelet-derived growth factor receptor alpha
Sox10: SRY-related HMG-box transcription factor 10.
